# Population genetics of the African snakehead fish *Parachanna obscura* along West Africa's water networks: Implications for sustainable management and conservation

**DOI:** 10.1002/ece3.9724

**Published:** 2023-01-16

**Authors:** Amien Isaac Amoutchi, Petra Kersten, Asja Vogt, Klaus Kohlmann, Essetchi Paul Kouamelan, Thomas Mehner

**Affiliations:** ^1^ West African Science Service Centre on Climate Change and Adapted Land Use (WASCAL) Graduate Research Program on Climate Change and Biodiversity Université Felix Houphouet‐Boigny Abidjan Côte d'Ivoire; ^2^ Leibniz‐Institute of Freshwater Ecology and Inland Fisheries Berlin Germany; ^3^ Laboratoire d'Hydrobiologie, UFR Biosciences Université Felix Houphouët Boigny Abidjan Côte d'Ivoire

**Keywords:** genetic differentiation, genetic diversity, hydrological networks, paleogeographic history, snakehead fish

## Abstract

An essential factor for aquatic conservation is genetic diversity or population divergence, which in natural populations reflects the interplay between geographical isolation with restricted gene flow and local evolution of populations. The long geological history of Africa may induce stronger among‐population divergence and lower within‐population divergence in fish populations of African watersheds. As an example, we studied population structure of the African snakehead fish *Parachanna obscura.* Our study aimed: (1) to develop a set of highly polymorphic microsatellite markers suitable for the analysis of genetic diversity among *P. obscura* and (2) to study the genetic diversity and structure of *P. obscura* populations from the West Africa region and mostly from Côte d'Ivoire, with respect to the effects of climate region and geographical distance on the genetic differentiation. A total of 259 specimens from 15 locations of *P. obscura* were collected over the West Africa region reflecting a high variability of pairwise geographical distances and variability of hydrological connectivity of the area. We developed a set of 21 polymorphic microsatellite markers for studying population genetics of the fish. The results showed relatively low intragenetic diversity for all the 15 locations, certainly attributable to confinement of fish in segregated catchments, resulting in limited gene flow. We also found evidence for local adaptation processes, suggested by five out of 21 microsatellite loci being under putative selection, according to BAYESCAN analysis. In turn, there was strong genetic differentiation (*F*_ST_ > 0.5) among fish from most locations, reflecting the allopatric development in watersheds without hydraulic connectivity. Neighbor‐joining dendrogram, Principal Coordinate Analysis, and analysis of ancestral groups by STRUCTURE suggested that the 15 locations can be divided into three clusters, mainly matching the dominant climate zones and the segregation of the watersheds in the region. The distinct genetic structure of the fish from the 15 locations obtained in this study suggests that conservation and sustainable management actions of this fish resource should avoid genetic mixing of potentially locally adapted populations.

## Graphical abstract

The long geological history of Africa may induce stronger among‐population divergence and lower within‐population divergence in fish populations of African watersheds. The study developed a set of 21 highly polymorphic microsatellite markers suitable for the analysis of genetic diversity among *Parachanna obscura*. We found relatively low intragenetic diversity and high genetic differentiation, resulting in limited gene flow, a dominance of local adaptation processes and an allopatric development in watersheds without hydraulic connectivity.

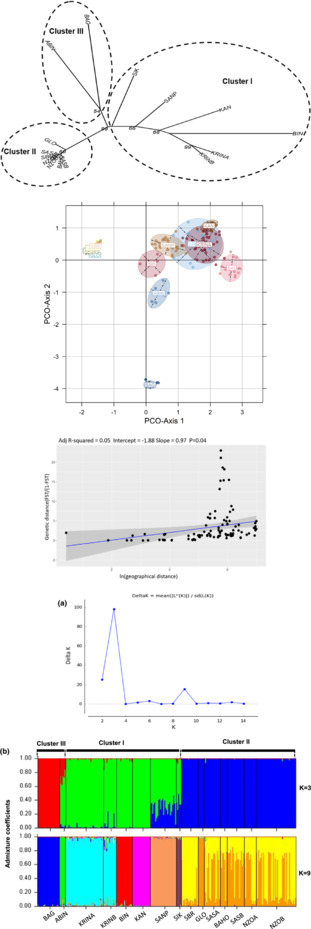

## 1 INTRODUCTION

Tropical regions host the world's richest freshwater fish faunas. According to estimates, there are over 3000 species of freshwater fish in Africa, which is comparable to the number of species in Asia (over 3600) and South America (over 4200; Lévêque et al., 2008). Complex climatic and geological events have caused a long history of geographical isolation followed by diversification for some and extinction for other populations, ultimately resulting in the diversified fauna of the African freshwater ecosystems (Amoutchi et al., 2021; Darwall et al., 2011). Since the division of Gondwanaland in the early Cretaceous, many African rivers went through complex geological histories involving changes in the structure of their catchments and river beds (Goudie, 2005). The evolution of Africa's fauna and landscapes has been influenced by the cycles between the Pleistocene and Pliocene dry and rainy eras (deMenocal, 2014; Maslin et al., 2014; Van Steenberge et al., 2020). Along with fluctuations of water levels in the African Great Lakes, these climate cycles have caused alternating expansions and contractions of savannah and forest‐like habitats (Malinsky & Salzburger, 2016). According to Tedesco et al. (2005), these climatic shifts led to migration, extinction, and allopatric divergence, which resulted in the current diverse fish faunas. In addition, the long geological history of Africa also affects the interplay between the contemporary distribution of populations and their genetic diversity, with longer periods of spatial segregation and allopatric development than those typically found for the much younger post‐glacial landscapes of the Palearctic or Nearctic.

An essential factor for aquatic conservation is genetic diversity or population divergence, which in natural populations reflects population history and the evolutionary potential of a species (Jaisuk & Senanan, 2018). Within a fish species, population subdivision results from the interaction of distinct genetic changes within isolated populations and restricted gene flow among them (Hedrick, 2011). Conspecific populations typically diverge from one another in the absence of gene flow due to mutation, natural selection, and genetic drift (Freeland, 2005). In addition, the degree to which landscape shapes patterns of genetic variation among populations is determined by life‐history features linked to migration and fish dispersal capabilities (Pilger et al., 2017). Geographical factors favoring population division include geographical distance between locations (Beneteau et al., 2009; Crookes & Shaw, 2016), the presence of barriers (Neville et al., 2006; Yamamoto et al., 2004), the complexity of a river network (Pilger et al., 2017), and habitat fragmentation (Sterling et al., 2012).

The freshwater ecosystem in Côte d'Ivoire in Africa is characterized by a large and complex system consisting of four major river basins: Sassandra, Cavally, Bandana, and Comeo ranging in length from 650 to 1160 km and rising in geographically wide‐ranging areas beyond the Côte d'Ivoire. In addition to these systems, there are many coastal rivers such as the Tabou, San Pedro, Niouniourou, Boubo, Agneby, Bia, and Me Rivers as well as two tributaries of the Niger River and many lakes (Girard et al., 1971). These habitats are endowed with numerous economically important fish species.

Among the economically important fish, *Parachanna obscura* (Gunther, 1861), is the most popular and widespread African fish species from the Channidae family. It is commonly known as African snakehead fish and has great economical and commercial values for local African communities. This species is benthopelagic and a strict freshwater habitant. It is generally distributed in the intertropical convergence zone where the water temperature ranges from 26°C to 28°C principally in West Africa. Nevertheless, it is also found in the upper course of the White Nile, the Lake Chad basin, and the Congo River basin (UA, 2013). Given the complexity and geological history of the region, the resulting patterns of genetic variation merit investigation in *P. obscura*. For example, Bezault et al. (2011) suggested that paleo‐geographic history, climatic events of Africa, and geographic barriers have induced strong genetic differentiation among *Oreochromis niloticus* populations from different parts of Africa. Strong genetic differentiation was also detected among African freshwater river and lake populations of *Lates niloticus*, reflecting the complexity of freshwater systems originating from the geological history of the continent (Basiita et al., 2018). Furthermore, the long‐time isolation of populations can favor adaptations to local environmental conditions with the occurrence of particular alleles. The adaptive capacity of populations depends on microevolution, e.g., the selection of local genotypes better adapted to changing environmental conditions (Canale & Henry, 2010; Hoffmann & Sgrò, 2011). These adaptation processes will likewise influence the amount and distribution of genetic diversity among populations (Pauls et al., 2013). Thus, assessment of the genetic diversity of *P. obscura* is necessary for understanding the evolutionary patterns of this species, and its capacity to cope with future environmental conditions, as well as for planning conservation policy for sustainable management of this species as fisheries resource.

Previous studies on *P. obscura* have mostly focused on biology (Bolaji et al., 2011; Odo et al., 2012), reproduction (Vodounnou et al., 2017), aquaculture potential (Azrita & Hafrijal, 2015), and phylogenetic range of the fish (Adamson et al., 2012; Conte‐grand et al., 2017; Li et al., 2003). However, there is no information about the genetic diversity among populations of this species. Since no previous studies have been conducted on the genetic diversity of *P. obscura*, genetic markers, for example, microsatellites, were not available. Accordingly, the objectives of our study were (1) to develop a set of highly polymorphic microsatellite markers suitable for the analysis of genetic diversity among *P. obscura* fish and (2) to study the genetic diversity and structure of *P. obscura* fish from 15 locations from the West Africa region and mostly from Côte d'Ivoire. According to the geological history of the region with potentially long periods of geographical isolation, we expected low genetic diversity and high genetic differentiation for this species. Furthermore, we expected that the genetic differentiation was affected by geographical isolation caused by the interplay between connectivity barriers, landscape structure, and geographical distances among locations.

## 2 MATERIALS AND METHODS

### 2.1 Study area and collection of samples

**FIGURE 1:**
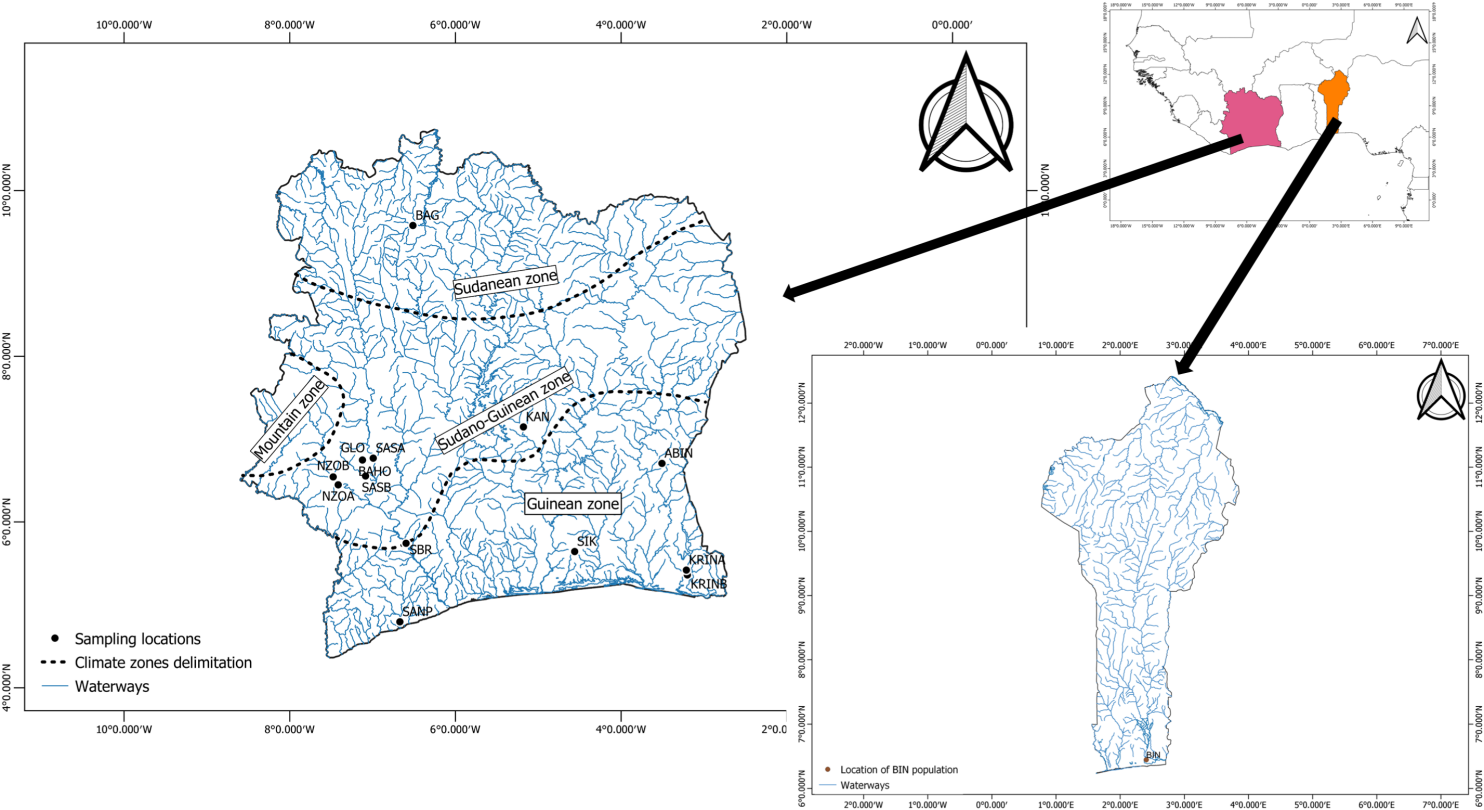
Map of the study area, showing sampling locations and abbreviations of locations (Table 1) from which *Parachanna obscura* specimens were collected, in Côte d'Ivoire (left) and in Benin republic (right down), and position of these countries in West Africa. The climate zones of Côte d'Ivoire are also shown.

A total of 259 specimens of *P. obscura* from 15 locations were collected over the West Africa region from Côte d'Ivoire (14 locations) and Benin republic (one location; see Figure 1 and Table 1). The sampling sites were selected to represent a high variability of pairwise geographical distances. In Côte d'Ivoire, individuals were sampled from Bia river (KRINA, and KRINB, locations about 7 km distant from each other), Wayadji stream (SIK), Sassandra river (SBR), San‐Pedro Lake (SANP), and Abengourou lake (ABIN). This region is characterized by a sub‐equatorial climate (Guinean climate zone) including two rainy and dry seasons, with an estimated annual precipitation of more than 1500 mm (Bernard, 2014). Specimens were also collected from Kan Lake (KAN), Baho (BAHO) and Glo (GLO) streams, Buyo Lake (SASA and SASB, sampling distance of about 26 km), and Nzo River (NZOA and NZOB, sampling distance of about 13 km), in the center and centre‐western Cote d'Ivoire, characterized by an equatorial transition climate (Sudano‐guinean climate zone), with two rainy and dry seasons, and an annual precipitation of 1200 to 1500 mm per year. Finally, individuals were collected in Bagoue river (BAG), situated in the north Cote d'Ivoire, characterized by a tropical climate (Sudanean climate zone) with a very hot and dry season from November to March and a rainy season from April to October. Specimens sampled from Benin republic were collected in Nokoue lake (BIN), located in the southern part characterized be a sub‐equatorial climate with two rainy and dry seasons.

**TABLE 1:** Information on samples sizes, basins, country, and climate of locations of 15 samples *Parachanna obscura* (with total sample size of 259 individuals).

River basin	Water body	Location	Sample size (*N*)	Country	Climate
Comoe	Abengourou Lake	ABIN	6	Côte d'Ivoire	Subequatorial
Coastal river Bia	Bia river	KRINA	38	Côte d'Ivoire	Subequatorial
Coastal river Bia	Bia river	KRINB	13	Côte d'Ivoire	Subequatorial
Coastal river San‐Pedro	San‐Pedro Lake	SANP	26	Côte d'Ivoire	Subequatorial
Sassandra	Sassandra river	SBR	17	Côte d'Ivoire	Subequatorial
Coastal river Agneby	Wayadji stream	SIK	5	Côte d'Ivoire	Subequatorial
Sassandra	Baho stream	BAHO	7	Côte d'Ivoire	Equatorial transition climate
Sassandra	Glo stream	GLO	6	Côte d'Ivoire	Equatorial transition climate
Bandama	Kan Lake	KAN	18	Côte d'Ivoire	Equatorial transition climate
Sassandra	Nzo river	NZOA	12	Côte d'Ivoire	Equatorial transition climate
Sassandra	Nzo river	NZOB	40	Côte d'Ivoire	Equatorial transition climate
Sassandra	Buyo Lake	SASA	16	Côte d'Ivoire	Equatorial transition climate
Sassandra	Buyo Lake	SASB	17	Côte d'Ivoire	Equatorial transition climate
Niger river	Bagoue river	BAG	22	Côte d'Ivoire	Tropical climate
Fed by Ouémé and Sô Rivers	Nokoue lake	BIN	16	Benin	Sub‐equatorial

*Parachanna obscura* fishermen from each sampling site were employed for fish samples collection using traps and fish nets. The intended sample size per location was 25–30 individuals; however, for several sites this number could not be obtained. For molecular analyses, approximately 1 cm of pectoral fin was clipped from each individual and stored in individual Eppendorf tubes containing 95% ethanol. The rest of the entire specimens were fixed in 10% formaldehyde solution and transported to laboratory for further analysis. The molecular analysis was performed in the genetic laboratory of the Leibniz‐Institute of Freshwater Ecology and Inland Fisheries, Berlin.

### 2.2 Molecular genetics analyses

Genomic DNA from fin clippings was isolated using the DNeasy Blood & Tissue Kit (Qiagen) following the manufacturer protocol. The development of microsatellite markers suitable for the analysis of population genetics of *P. obscura* was conducted by the commercial company GenoScreen. Their procedure consisted of two steps: (1) GenoSat library preparation using 5 μg DNA from an equimolar pool of 10 DNA samples followed by high throughput DNA sequencing run on Nano 2x250 v2—MiSeq Illumina and bioinformatic analysis and primer design. (2) Biological validation of 142 primer pairs on eight *P. obscura* DNA samples from different populations including PCR amplification and analysis of the obtained profiles on QIAxcel (Qiagen). GenoScreen usually considers as validated those primer pairs with a specific PCR product at the expected size for at least 5 samples. This was the case for 120 (or 84.5%) of the tested 142 primer pairs. Based on the delivered list of validated primers with comments for each pair and migration profiles generated by QIAxcel ScreenGel 1.6.0, the number of potentially suitable primer pairs was further reduced by excluding those with weak, very weak, or no visible PCR product for one or more samples and focusing on those indicating polymorphism on the migration profiles. This screening resulted in 29 primer pairs selected for testing on a larger number of individuals using the PCR protocol described below. However, only 21 primer pairs (Appendix A) turned out to be suitable for routine genotyping based on the observed polymorphism.

Each forward primer of the 21 loci was synthesized with either DY751 or Cyanine 5 or BMN‐6 fluorescent dyes attached to its 5′ end. A set of eight multiplex reactions were conducted (Appendix B). PCR amplification was conducted using Qiagen Multiplex PCR kits, following the recommendations of the manufacturer. PCR amplification was carried out in 11.3 μl reaction volumes, containing 10 μl PCR mix and 1.3 μl DNA (~ 20 ng/μl). The composition of the PCR mix of each of the multiplex reactions is described in Appendix B. The primer concentration was 10 pmol/μl. The thermocycling profile started with an initial denaturation step at 95°C for 15 min, followed by 35 cycles of 30 s at 94°C, 55°C, and 72°C and ended with a final extension step of 30 min at 60°C. Denatured fragments were resolved on an automated DNA sequencer (Beckmann Coulter CEQ 8000) using 0.83 μl of PCR product and a mixture of 30 μl formamide (SLS; Sciex) and 0.5 μl size standard‐400 (Sciex). Genotypes were identified using the GenomeLabTM GeXP Genetic Analysis System version 10.2 (Beckman Coulter) fragment analysis module.

### 2.3 Statistical data analyses

The majority of the analyses and graphical output were created using R 4.1.2 (R Core Team, 2021). Allele polymorphism at each of the 21 microsatellite loci and the intra‐population genetic diversity metrics, such as number of alleles per location and allelic richness A_R_, were quantified by PopGenReport 3.0 package (Adamack & Gruber, 2014). Potential existence of individuals with missing genotypes among the loci was evaluated using poppr 2.9.3 (Kamvar et al., 2014) package in R. Null allele frequencies per locus were calculated in the genepop 1.1.7 package (Rousset, 2008). The inbreeding coefficient (probability that the two alleles at one locus of an inbred individual are identical alleles per descent (Gazal et al., 2014); *F*_IS_) was estimated in genepop_in_R. The significance of this coefficient was evaluated based on 95% confidence intervals calculated using bootstrapping (*N* = 100) in hierfstat 0.5–11 package in R (Jerome & Thibaut, 2022). Deviations from Hardy–Weinberg equilibrium (HWE) at the location level were tested by the exact (probability) test using genepop v1.1.7 in R. Probabilities of HWE deviations (Guo & Thompson, 1992) per locus per location were corrected by the false discovery rate for multiple tests (Benjamini & Hochberg, 1995), supplemented by U‐tests on excess homozygotes. We conducted an Analysis of Molecular Variance (AMOVA, Excoffier et al., 1992) using the R‐package pegas 1.1 (Paradis, 2010) to compare within‐ and between‐location variance for the 15 locations.

Through the genepop v1.1.7 package in R, genetic differentiation was estimated by *F*‐statistics between locations (*F*_ST_; Weir & Cockerham, 1984), with the significance of differentiation assessed by exact conditional contingency table tests for genotypic differentiation. Using the poppr v2.9.3 package in R, a neighborhood tree (Saitou & Nei, 1987) based on Prevosti's pairwise genetic distance (Prevosti, 1974) was constructed to identify genetic relationships between *P. obscura* locations. The 259 individual genotypes were also subjected to a principal coordinate analysis (PCoA), which is a multidimensional metric scaling, in order to visualize the distribution of individuals and location centroids in reduced space. This analysis was carried out in ade4 v1.7–19 (Thioulouse et al., 2018), while adegraphics v 1.0–16 (Siberchicot et al., 2017) was used to visualize the resulting graphs.

Bayesian approaches as implemented in the standalone program STRUCTURE v2.3.4 (Pritchard et al., 2000) were used to estimate and visualize the genetic structure among samples from the 15 locations. After a burn‐in period of 100,000 iterations, the number of Markov‐chain Monte Carlo iterations was set to 100,000 using admixture model's default parameters and locations' correlated allele frequencies. Ten runs were performed for each K (ancestral groups or clusters) between 1 and 15. The greatest mean estimate of the posterior probability based on the ad hoc ΔK statistics was used to determine the optimum K (Evanno et al., 2005).

Mantel correlation tests were performed between the pairwise genetic distance matrices calculated as *F*_ST_/(1‐*F*_ST_) values and the natural logarithm (ln) of the pairwise straight‐line geographic distances (km) to assess the effect of geographic isolation or distance on the genetic structure among *P. obscura* sample locations. The genetic distance matrix was calculated in the genepop package of R, while the geographical distance matrix was calculated with QGIS 3.22.6 using the global positioning system (GPS) coordinates of the sampling locations. Mantel correlation tests with 1000 permutations were performed by the vegan v 2.6–2 package (Oksanen et al., 2022) in R.

To examine non‐neutral evolutionary forces acting on the microsatellite loci, a scanning analysis was realized using the BAYESCAN v2.1 software (Foll & Gaggiotti, 2008) to detect candidate loci under selection. BAYESCAN was run with a sample size of 5000, a number of pilots runs of 20, length of pilot runs of 5000, a burn‐in of 50,000, and the false discovery rate (FDR) threshold of 0.05.

## 3 RESULT

### 3.1 Genetic structure within populations

In total, 0.3% missing genotypes were obtained among all microsatellites for the 259 individuals from the 15 locations (Appendix C). Loci Para041 (1.9%) and Para023 (1.5%) had the highest proportions of missing genotypes. Among locations, ABIN (1.6%) and SIK (1.0%) recorded the highest proportions of missing loci. No individual with more than two missing loci was found. Therefore, all individuals and loci were used for further analyses.

A total of 87 alleles were observed across the 21 microsatellite loci. All microsatellite markers developed in this study were variable. The allele number (*N*_A_) per locus ranged from two found at Para038, Para128, Para136, and Para137 loci to 12 found at Para027 locus (Appendix D). Among the loci genotyped, Para027 was the most variable one (*H*_E_ = 0.67, *H*_O_ = 0.42), while the lowest variability was detected at locus Para042 (*H*_E_ = 0.21, *H*_O_ = 0.03). Following maximum likelihood estimates (Dempster et al., 1977), relatively high null allele frequencies were estimated for loci Para038 (16%) and Para059 (59%). However, since no difference was observed in the estimates of genetic differentiation among locations after removing these loci (pairwise *F*_ST_ values; Appendix E), we included all 21 loci in the analyses.

The genetic diversity was low in most of the locations (Table 2). However, KRINA (*N*_A_ = 57, *H*_O_ = 0.35, *H*_S_ = 0.33, *Ᾱ*_*R*_ = 1.9), KRINB (*N*_A_ = 55, *H*_O_ = 0.33, *H*_S_ = 0.34, *Ᾱ*_*R*_ = 2.0) and BIN (*N*_A_ = 57, *H*_O_ = 0.302, *H*_S_ = 0.30, *Ᾱ*_*R*_ = 1.9) had a higher genetic diversity than the other locations. The estimated inbreeding coefficient (*F*_IS_) value was low for all locations, and the confidence intervals showed that the *F*_IS_ coefficients were non‐significant for all locations. Except for SASA (Para 107), SASB (Para027) and KRINA (Para104) locations where deviations from HWE at single loci were found (Appendix F), most of the samples did not deviate from HWE for all 21 analyzed microsatellite loci after correction by the false discovery rate for multiple tests.

**TABLE 2:** Genetic diversity of *Parachanna obscura* fish from 15 sampling locations at 21 microsatellite loci

Location	*N*_A_	*H*_O_	*H*_S_	*Ᾱ* _R_	*F*_IS_	95% CI for *F*_IS_
ABIN	33	0.16	0.18	1.4	0.09	[−0.17; 0.33]
BAG	27	0.02	0.023	1.1	−0.04	[−0.06; 0.00]
BAHO	23	0.02	0.03	1.1	0.28	[0.00; 0.37]
BIN	57	0.30	0.30	1.9	−0.01	[−0.15; 0.14]
GLO	22	0.02	0.02	1.0	−0.25	[−0.25; −0.25]
KAN	28	0.09	0.10	1.2	0.09	[−0.05; 0.22]
KRINA	57	0.35	0.33	1.9	−0.06	[−0.14–0.01]
KRINB	55	0.33	0.34	2.0	0.01	[−0.08; 0.13]
NZOA	23	0.04	0.04	1.1	0.07	[−0.22; 0.29]
NZOB	25	0.04	0.04	1.1	0.09	[0; 0.11]
SANP	35	0.27	0.26	1.6	−0.05	[−0.22; 0.12]
SASA	25	0.04	0.05	1.1	0.19	[−0.09; 1]
SASB	25	0.02	0.03	1.1	0.40	[−0.03; 0.54]
SBR	24	0.06	0.05	1.1	−0.16	[−0.19; −0.13]
SIK	34	0.30	0.26	1.6	−0.14	[−0.41; 0.13]

Abbreviations: 95% CI, 95% confidence interval; *F*_IS_, inbreeding coefficient; H_O_, observed heterozygosity_;_ H_S_, expected heterozygosity; N_A_, Observed number of alleles; Ᾱ_R_, Mean allelic richness.

### 3.2 Genetic structure between populations

The pairwise *F*_ST_ was high and significantly differentiated all location pairs of *P. obscura*, except the few locations from the same watershed only few kilometers apart (Table 3). BAG was most genetically differentiated, with the highest pairwise *F*_ST_ values (all above 0.7) recorded relative to the other location pairs. Estimation of the pairwise *F*_ST_ was extremely low (close to zero) between SASA, SASB, NZOA, NZOB, and BAHO from Sassandra river basin and between KRINA and KRINB from Bia river.

**TABLE 3:** Matrix of pairwise F_ST_ between the 15 *P. obscura* locations (lower diagonal), and their *p*‐values as obtained by G‐tests (upper diagonal)

	ABIN	BAG	BAHO	BIN	GLO	KAN	KRINA	KRINB	NZOA	NZOB	SANP	SASA	SASB	SBR
ABIN		**<0.0001**	**<0.0001**	**<0.0001**	**<0.0001**	**<0.0001**	**<0.0001**	**<0.0001**	**<0.0001**	**<0.0001**	**<0.0001**	**<0.0001**	**<0.0001**	**<0.0001**
BAG	**0.90**		**<0.0001**	**<0.0001**	**<0.0001**	**<0.0001**	**<0.0001**	**<0.0001**	**<0.0001**	**<0.0001**	**<0.0001**	**<0.0001**	**<0.0001**	**<0.0001**
BAHO	**0.81**	**0.95**		**<0.0001**	**0.0255**	**<0.0001**	**<0.0001**	**<0.0001**	0.388	0.469	**<0.0001**	0.755	1	**0.006**
BIN	**0.67**	**0.82**	**0.72**		**<0.0001**	**<0.0001**	**<0.0001**	**<0.0001**	**<0.0001**	**<0.0001**	**<0.0001**	**<0.0001**	**<0.0001**	**<0.0001**
GLO	**0.80**	**0.96**	**0.66**	**0.73**		**<0.0001**	**<0.0001**	**<0.0001**	**0.0005**	**<0.0001**	**<0.0001**	**<0.0001**	**0.0009**	**<0.0001**
KAN	**0.79**	**0.93**	**0.85**	**0.63**	**0.85**		**<0.0001**	**<0.0001**	**<0.0001**	**<0.0001**	**<0.0001**	**<0.0001**	**<0.0001**	**<0.0001**
KRINA	**0.56**	**0.72**	**0.52**	**0.38**	**0.53**	**0.42**		0.277	**<0.0001**	**<0.0001**	**<0.0001**	**<0.0001**	**<0.0001**	**<0.0001**
KRINB	**0.56**	**0.80**	**0.55**	**0.41**	**0.54**	**0.50**	0.01		**<0.0001**	**<0.0001**	**<0.0001**	**<0.0001**	**<0.0001**	**<0.0001**
NZOA	**0.83**	**0.95**	0.04	**0.74**	**0.61**	**0.85**	**0.54**	**0.59**		0.999	**<0.0001**	0.608	0.244	0.054
NZOB	**0.88**	**0.94**	0.07	**0.83**	**0.59**	**0.88**	**0.64**	**0.72**	−0.03		**<0.0001**	0.223	**0.027**	**0.037**
SANP	**0.58**	**0.78**	**0.51**	**0.54**	**0.56**	**0.53**	**0.34**	**0.34**	**0.53**	**0.63**		**<0.0001**	**<0.0001**	**<0.0001**
SASA	**0.83**	**0.94**	−0.02	**0.76**	**0.55**	**0.85**	**0.56**	**0.61**	0.01	0.05	**0.54**		0.478	**0.018**
SASB	**0.85**	**0.95**	−0.07	**0.78**	**0.61**	**0.87**	**0.57**	**0.63**	0.07	**0.11**	**0.57**	0.00		**<0.0001**
SBR	**0.84**	**0.94**	**0.22**	**0.76**	**0.61**	**0.85**	**0.56**	**0.61**	0.08	**0.09**	**0.53**	**0.11**	**0.25**	
SIK	**0.56**	**0.89**	**0.68**	**0.59**	**0.65**	**0.68**	**0.47**	**0.45**	**0.71**	**0.80**	**0.46**	**0.72**	**0.76**	**0.70**

*Note*: Significant pairwise differentiation indicated in bold.

The result of AMOVA showed significantly (*p* < .05) high genetic variation among locations (66.7%) and within individuals (33.5%), while no significant variance was detected among individuals within locations (−0.2%; Table 4).

**TABLE 4:** Analysis of molecular variance for 259 *Parachanna obscura* individuals originating from 15 locations sampled for this study

	df	Sum Sq	Mean Sq	Sigma	% of variation	*p*
Variance between locations	14	2958.9	211.3	6.2	66.7	.01
Variance between samples within locations	244	747.6	3.1	−0.02	−0.2	.59
Variance within individuals	259	805.0	3.1	3.1	33.5	.01
Total variance	517	4511.5	8.7	9.3	100	

**FIGURE 2:**
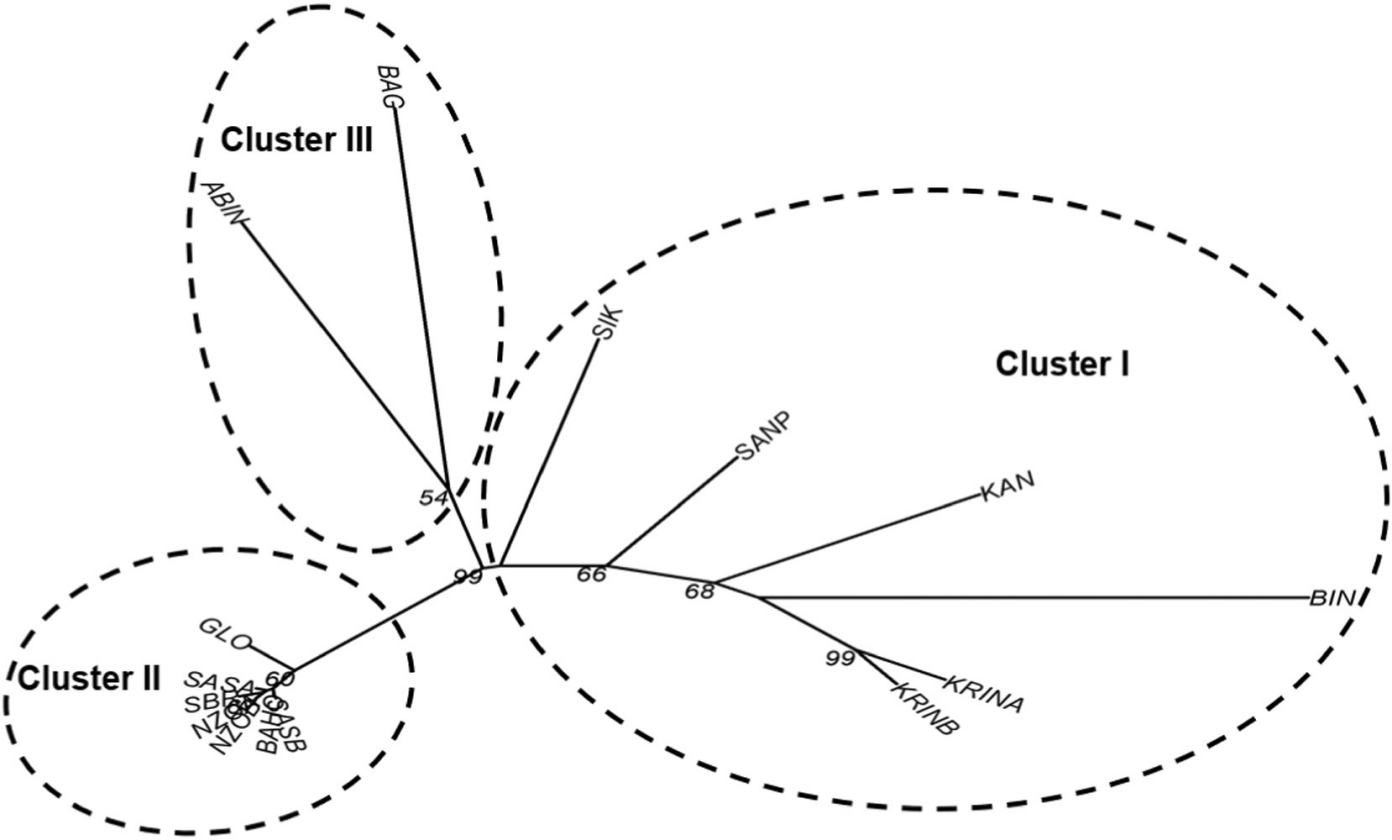
Neighbor‐joining dendrogram of *Parachanna obscura* fish from 15 sampling locations, based on Prevosti's genetic distance. Only bootstrap values >50% are shown.

**FIGURE 3:**
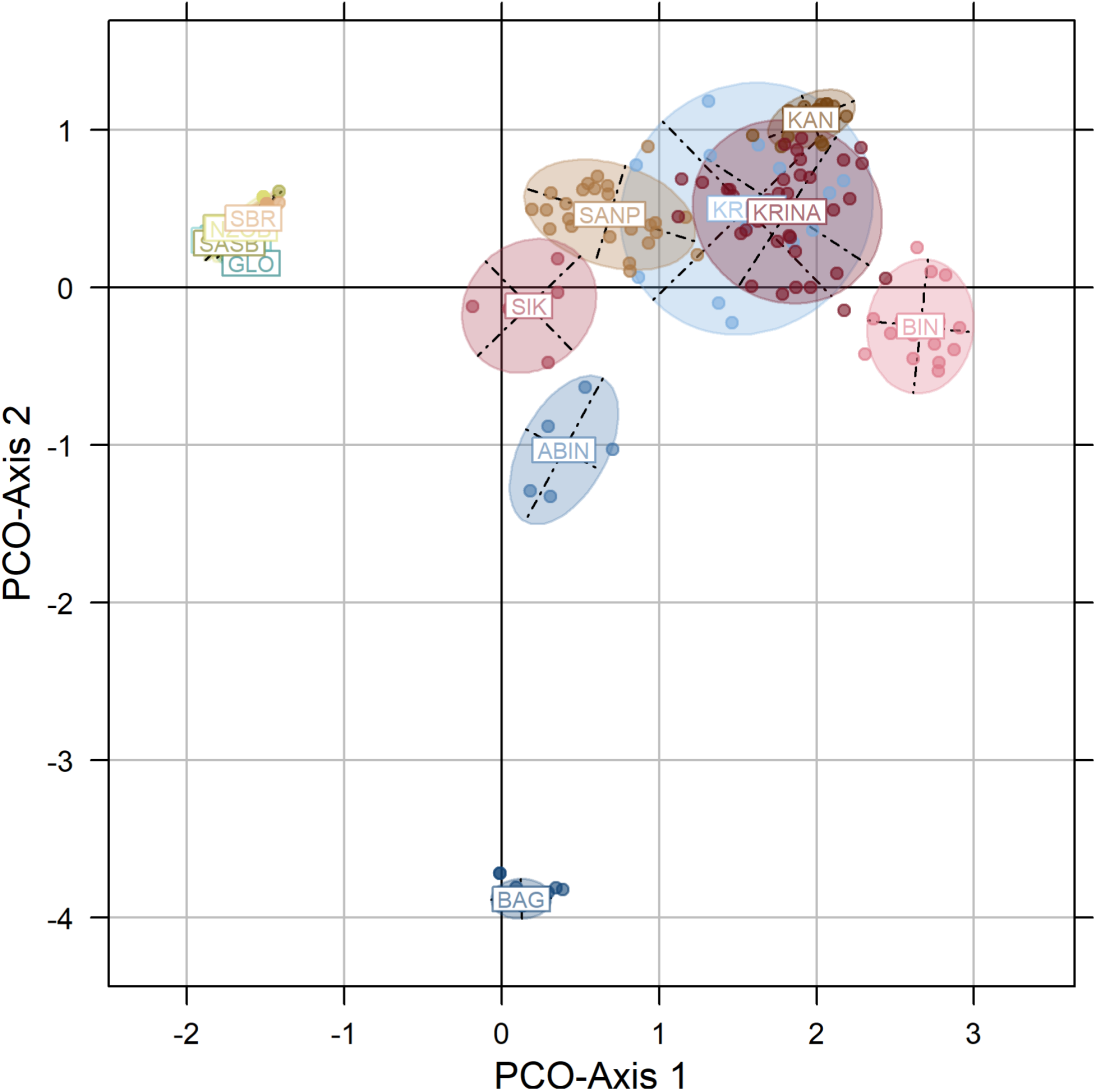
Axes 1 and 2 of the principal component analysis (PCoA) of 259 *Parachanna obscura* individuals (dots) and inertia ellipses representing 15 sampling locations.

The neighbor‐joining dendrogram obtained based on Prevosti's genetic distance separated the locations into three major clusters (Figure 2). The locations SIK, KRINA, KRINB, SANP, KAN, and BIN fish representing cluster I are located in southern regions characterized by sub‐equatorial climate, except KAN from the central Côte d'Ivoire. The second cluster (II) was composed of BAG (Bagoue river) and ABIN (Abengougou lake) from northern (with a tropical climate) and south‐eastern Côte d'Ivoire (with sub‐equatorial climate), respectively. The third cluster (III) included SASA, SASB, NZOA, NZOB, GLO, SBR, and BAHO locations fish from central‐western region of Côte d'Ivoire with an equatorial transition climate. Principal coordinate analysis (PCoA) of individuals confirmed the classification of locations obtained from the dendrogram (Figure 3). The first five axes explained around 77.4% of the total predicted variation with 36.9%, 21.7%, 7.5%, 5.8%, and 5.3% of predicted variation, respectively. The projection of the 259 specimens over the first two axes is shown in Figure 3. PCoA‐Axis 1 principally separated samples of cluster II (negative PCoA‐values) and samples of cluster I (positive PCoA‐values). PCoA‐Axis 2 isolated cluster III locations BAG and ABIN with negative PCoA‐values from the other locations.

**FIGURE 4:**
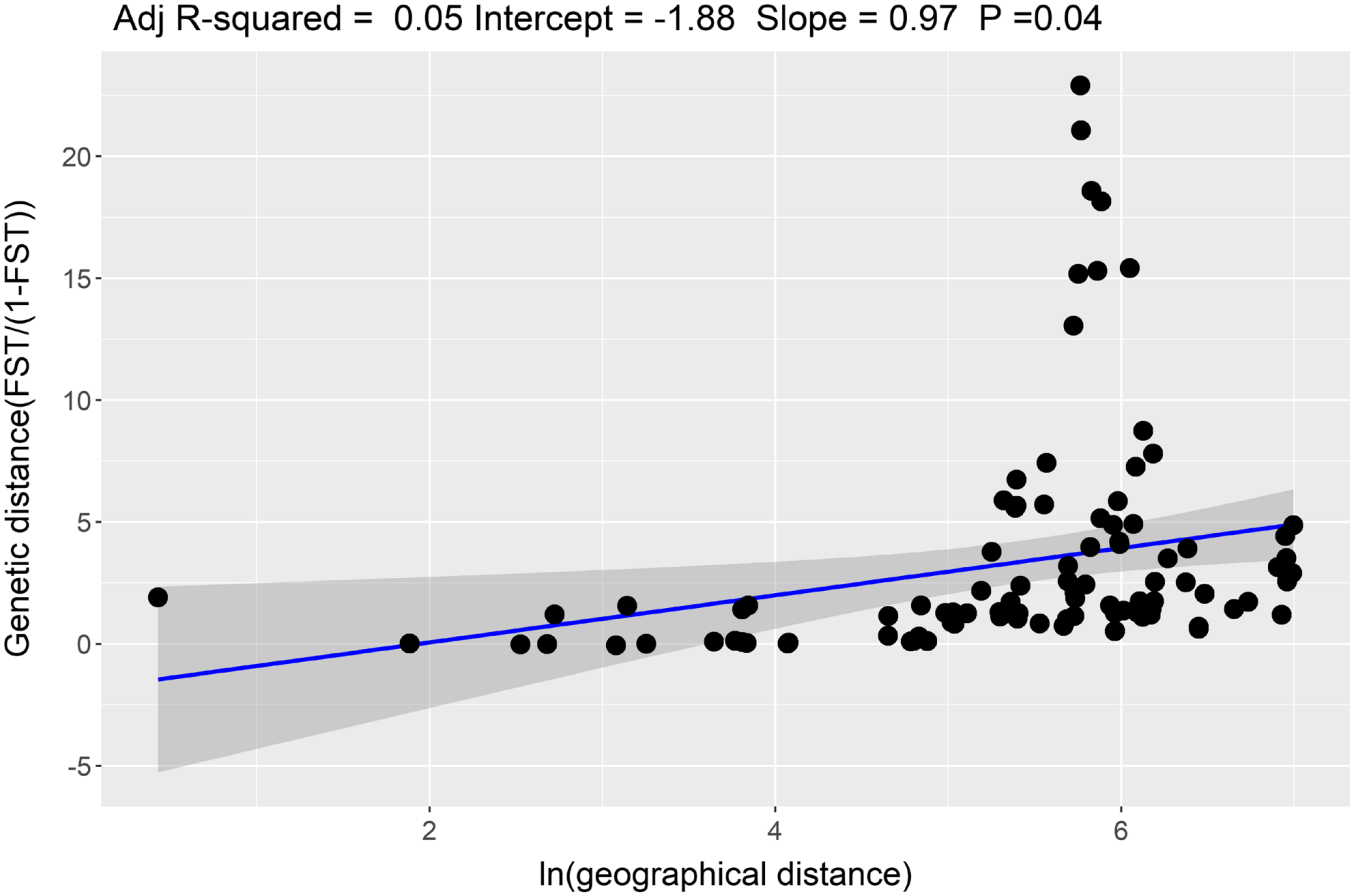
Multilocus estimates of pairwise genetic distances (*F*_ST_/(1−*F*_ST_)) between fish from 15 sampling locations plotted against natural logarithm (ln) of pairwise Euclidean geographical distance (km). *p* was obtained based on Mantel test.

A complex spatial genetic structure of *P. obscura* samples was revealed by the correlation between pairwise genetic distances (*F*_ST_/(1‐*F*_ST_)) and the natural logarithm of the pairwise Euclidean geographical distance between the 15 locations. Although there was a significant positive trend between genetic and geographical distances, there were strong deviations from the linear pattern at 5.5 and 6.5 ln geographical distances, caused by the strong genetic distances between BAG and SASA, SASB, NZOA, NZOB, BAHO, and BAHO locations (Slope = 0.96, Adj *R* ^2^ = 0.052; Mantel test *p* = .04, Figure 4).

**FIGURE 5:**
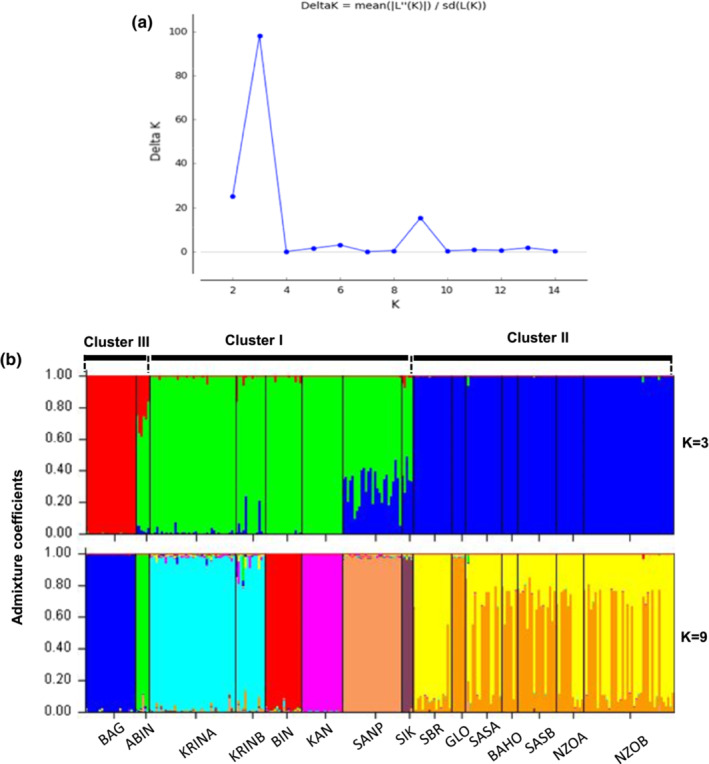
Admixture analysis by STRUCTURE of *Parachanna obscura* fish obtained from 15 sampling locations. (a) Line plot of successive ΔK from *K* = 2–14. (b) Bar plots of genetic clusters for *K* = 3 and *K* = 9 ancestral groups, where each color corresponds to a unique ancestral group. Markov‐chain Monte Carlo iterations were set at 100,000 after a burn‐in period of 100,000.

The fish from the 15 locations could be divided into three ancestral genetic groups (Figure 5), following the peak value Δ*K* ~ 100 (Figure 5a Evanno et al., 2005). However, a second small peak of Δ*K* (~18) was recorded at *K* = 9, suggesting that nine ancestral groups would be an alternative splits worth exploring. With *K* = 3, the STRUCTURE clustering diagram corresponded to differentiation among locations into three clusters as proposed previously in the distance‐based and Principal coordinate analysis. However, individuals of SANP and SIK locations displayed a more complex genetic structure with shared membership of ancestral origins from clusters I and II. Also, ABIN defined as belonging to cluster III shared memberships with clusters I and II. Considering *K* = 9, the STRUCTURE clustering diagram showed that the locations of the previously defined cluster II were clearly differentiated from the other locations, with two admixed ancestral genetic groups (yellow and orange). The other locations were unique with dominance of one ancestral group in each location, except KRINA and KRINB which belong to the same ancestral group.

### 3.3 Detection of loci potentially under selection

**FIGURE 6:**
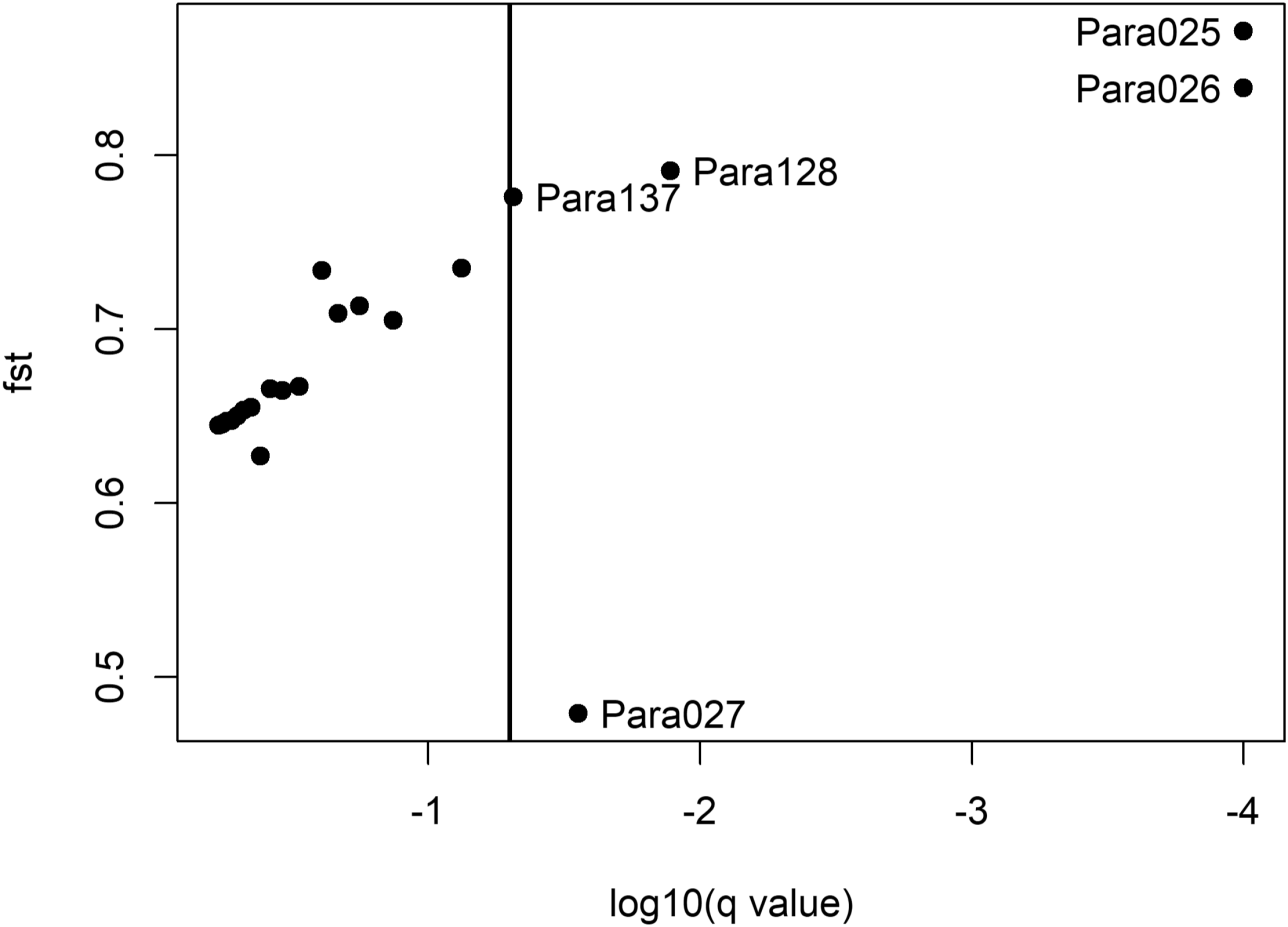
Plot representing the BAYESCAN results searching candidate loci under selection. The vertical line represents a false discovery rate (FDR) threshold of 0.05. Points to the right of the vertical line represent loci under selection. *Q*‐value: Minimum false discovery rate at which a locus may become significant; *F*_ST_, Coefficient to measure the difference in allele frequency between the common gene pool and each population, calculated as a posterior mean using model averaging.

The BAYESCAN analysis indicated five among the 21 loci with a q‐value of less than 5%, suggesting that they are under selection (Figure 6). Of these five loci, Para027 with lowest *F*_ST_ and negative alpha (coefficient indicating the strength and direction of selection) was putatively under balancing selection, while the other four loci (with highest *F*_ST_ and positive alpha) were under putatively directional selection (see Appendix G). However, the *F*_ST_ estimates showed high values for all loci (>0.4), also for those with *q* > 0.05 (Figure 6).

## 4 DISCUSSION

We characterized the genetic structure and diversity of *P. obscura* fish from 15 locations in the West Africa region (mostly Côte d'Ivoire), using the set of polymorphic microsatellite markers developed in the current study. Among the 120 produced and validated markers, a group of 21 polymorphic microsatellites were deemed suitable for the analysis of genetic differentiation among the sampling locations. However, some of these markers had a slightly increased frequency of null alleles. Microsatellite null alleles have been documented in studies of population genetics and PCR primer characterization (Dakin & Avise, 2004) and have been discovered in a wide variety of taxa. Examples include insects (Lepidoptera, reviewed in Meglecz et al., 2004; Diptera, Lehmann et al., 1997; and Orthoptera, Chapuis et al., 2005) and mollusks (Astanei et al., 2005; Li et al., 2003). It is generally not advised to include null allele loci in population genetics because they may affect estimates of population differentiation (Chapuis & Estoup, 2007). However, we did not exclude any locus from our study because it has been demonstrated that the influence of null alleles in studies of population genetics may be minimal compared to other parameters such the number of loci (Carlsson, 2008). Moreover, for our data, the estimation of the populations differentiation (*F*_ST_) using only 19 microsatellites showed no difference in the value compared to the original data including null alleles. Jaisuk and Senanan (2018) also found no change in population differentiation of *Garra cambodgiensis* after removing alleles with high null alleles frequencies.

The estimated within‐population genetic diversity was low for all the 15 locations. In contrast to our result, high genetic diversity was obtained in other fish species occurring in Africa and other parts of the world using microsatellite loci. Angienda et al. (2011) reported a relatively high genetic diversity for *Oreochromis esculentus* (*H*_O_ = 0.795–0.81, *H*_S_ = 0.745–0.77, *Ᾱ*_*R*_ = 7.13–8.13) and *Oreochromis niloticus* (*H*_O_ = 0.70–0.78, *H*_S_ = 0.71–0.79, *Ᾱ*_*R*_ = 7–8.38) populations in eastern Africa. Mehner et al. (2021) found high genetic diversity in the European *Coregonus* cisco populations (*A*_*R*_ = 3.4–6.3). Many studies on the genetic diversity of other species from the Channidae family such as *Channa striata* (Adamson et al., 2012; Robert et al., 2019; Tan et al., 2016) and *Channa marulia* (Pathak et al., 2018) showed relatively high genetic diversity. The low within‐population genetic diversity observed in our study is likely a result of limited gene flow among populations. Also, the very low genetic diversity observed in SASA, SASB, NZOA, NZOB, and GLO, all originating from the same basin (Sassandra river basin), may be due to the Wahlund effect, which occurs when genotypic proportions are computed from heterogeneous samples where individuals belonging to genetically differentiated entities (subpopulations) are pooled (De Meeûs, 2018), resulting in an excess of homozygotes. This assumption is supported by the STRUCTURE results (*K* = 9, Figure 5) with the mix from two ancestral groups in these samples, as well as the positive *F*_IS_ obtained for these samples. Furthermore, confinement of populations in segregated catchments have probably facilitated local adaptation, thus further reducing genetic diversity. Microsatellite markers are normally neutral to selection. However, adaptive micro‐evolutionary mechanisms may reduce genetic diversity both at selected loci under adaptation and in those parts of the genome that are hitchhiking with them (Via, 2009; Via & West, 2008). Genetic hitchhiking is a process that allele frequencies change without being under natural selection, because these genes are located on the same DNA chain near to another gene that is undergoing a selective sweep. The hitchhiking process may affect larger regions of the genome in particular in small populations (Charlesworth et al., 1997). As a result, genetic diversity of populations may decrease fast in response to environmental changes, lowering the population's ability to respond to future selection pressures. We found high *F*_ST_ for all loci and five loci suggested to be under selection in the BAYESCAN analysis, indicating that micro‐evolutionary mechanisms may have contributed to the reduction of the genetic diversity.

The estimation of the pairwise genetic differentiation (*F*_ST_) showed highly significant differences among most of the locations of *P. obscura* except among locations from connected river networks. This result suggests that the fish from most of the different locations belong to distinct populations, except those collected from interconnected water networks, which may form one unique population together. In our study, SASA, SASB, NZOA, NZOB, and BAHO are samples from interconnected rivers (Nzo River, Baho and Glo streams) and therefore had low values of pairwise genetic differentiation (*F*_ST_ close to zero). Nzo River, Baho and Glo streams are tributaries of Buyo lake, which is an artificial lake created by dam construction on Sassandra river. Thus, fish from these five locations can be considered as belonging to one population. KRINA and KRINB are both from Bia River, with samplings only a few km apart, and therefore likewise can be considered belonging to the same population.

Aquatic species, and freshwater fishes in particular, frequently display strong population structure probably as a result of their confinement to the network structure of aquatic systems in the landscape (Loxterman, 2011; Pérez‐Espona et al., 2008). The pattern of genetic differentiation obtained in our study suggests an allopatric evolution of populations of *P. obscura*. The freshwater systems of the area where populations were collected are heterogeneous and complex, characterized by geographical barriers and lack of connectivity, which has favored the isolation of populations and prevented gene flow. Isolation of fish populations based on height of watershed boundaries and relatively frequent occurrence of movement barriers within watersheds, have generally been demonstrated to generate significant genetic differentiation (Faulks et al., 2010; Gomez‐Uchida et al., 2009; Loxterman & Keeley, 2012; Pfrender et al., 2004; Taylor et al., 2003). The results are in line with the hypothesis developed by Meffe and Vrijenhoek (1988) for explaining population genetic patterns for aquatic organisms inhabiting stream networks. Indeed, these authors predict that genetic isolation will occur among stream networks that do not have hydrological connections, resulting in an imbalance between drift and gene flow (where gene flow is effectively zero). A study on *Channa argus*, another Channidae species, revealed significant genetic differentiation among populations related to the structure of the river system (Yan et al., 2018). Loxterman and Keeley (2012) also demonstrated that watershed boundaries have probably driven the genetic isolation obtained among Cutthroat trout (*Oncorhynchus clarkii*) populations from western North America.

The high values of the genetic differentiation obtained between locations from unconnected water networks (*F*_ST_ higher than 0.5 in most cases) and the high genetic variation obtained among locations (66.7%) from AMOVA in our study, suggest that the populations have been isolated for long period of time, probably matching with the paleogeographic history of the formation of the hydrological systems on the African continent. Indeed, various paleogeographic (such as the disruption of fluvial drainages) and paleoclimatic (such as wet‐dry cycles) events have had a significant impact on the African ichthyofauna from the Miocene to the Pleistocene by altering the connectivity between the various hydrographic systems (Bezault et al., 2011; Drake et al., 2011; Lévêque, 1997). A study by Bezault et al. (2011) demonstrated that paleo‐geographic events have resulted in high genetic differentiation among *Oreochromis niloticus* populations across Africa. Using mitochondrial DNA based markers, Mwanja et al. (2013) found different lineages of *Lates* sp, which may have developed during geographical isolation during the Pleistocene and have remained largely allopatric without gene flow since that time, on the African continent. The effect of paleo‐historical lake level variations on genetic diversity of African cichlids has also been demonstrated (Egger et al., 2007).

Although there was a significant and positive trend between genetic and geographical distances, some location pairs strongly deviated from the expected linear pattern. This pattern suggests that genetic diversity may additionally be shaped by insurmountable barriers resulting from landscape characteristic between the different sampling locations that prevent any exchange of genetic material among populations from non‐connected water bodies. The population most strongly differentiated from all others was from Bagoue River (BAG), situated in the north Cote d'Ivoire, characterized by a tropical climate. As seen on the map, the watersheds of Bagoue River are completely disconnected from the watersheds of the other populations analyzed, making geographical separation very likely. This separation is likely induced by the geography of the area, which is characterized by a mountain ridge south of Bagoue River that prevents the river flowing to the south, as most of the other rivers in the southern part. Similar observations were taken on salmonid fishes for which it has been suggested that landscape characteristics such as the complexity of the drainage network and differences in channel gradients between habitats are likely to limit dispersal between their populations (Angers et al., 1999; Castric et al., 2001; Guy et al., 2008; Hebert et al., 2000).

The classification of *P. obscura* populations into three clusters from both the neighbor‐joining dendrogram and the principal coordinate analysis suggested that the genetic diversity of the populations may have probably been shaped by isolation due to geographical barriers and micro‐evolutionary adaptive mechanisms, with climate‐related environmental conditions as a potential driver. Indeed, the location of the populations in these clusters partly matched the climatic zones of Ivory coast and Benin republic. In the first cluster, five populations are combined, which originate from regions with a sub‐equatorial climate. Only fish from the KAN location were collected in an area characterized by an equatorial transition climate. The second cluster was composed of samples from an equatorial transition climate, except the SBR. SBR is linked to other populations of this cluster by habitat connectivity which facilitate gene flow. The third cluster included two populations, with BAG located in the tropical climate regions and ABIN located in sub‐equatorial climate regions. The population structure characterized by the ancestral groups inferred by admixture coefficients in STRUCTURE has given the same classification (*K* = 3) but showed that ABIN shared a higher admixture with those populations from the sub‐equatorial climate areas, which form cluster I. Therefore, it is more likely that ABIN location also belongs to cluster I. The partly match between clusters and climate zones suggests that regional climate conditions may have contributed to the patterns of genetic diversity of *P. obscura* in West Africa. However, more appropriate types of markers (e.g., SNP data obtained through RAD‐seq) should be applied in future for confirming this hypothesis of regional climate as driver of the pattern of genetic diversity in *P. obscura*. In contrast, the population structure at higher resolution with classification into nine ancestral groups (*K* = 9) reflects the geographical configuration of the network of watersheds with populations from the same watersheds belonging to the same cluster. Hence, hydraulic connectivity or isolation have shaped gene flow among the habitats, facilitating differing evolution of the populations.

## 5 CONCLUSIONS

Using the collection of polymorphic microsatellite markers developed in the present work, we characterized for the first time, the genetic diversity, and structure of *P. obscura* populations from West Africa, representing an important baseline for further exploration of the population dynamics in this species. Understanding the genetic diversity of wild populations can help establishing aquaculture breeding programs as well as conservation initiatives to preserve fish stocks and their unique genetic identities (Yan et al., 2018). The low genetic diversity of *P. obscura* demonstrated that particular attention has to be paid for conservation and sustainable management of this fish resource. Regarding the high genetic differentiation between populations attributable to habitat heterogeneity and local adaptation, in situ conservation will be required in order to maintain genetic integrity. These population may serve as reservoirs or stocks for future selection programmes in aquaculture as well as improving population fitness and ability to respond to future environmental disturbance.

## AUTHOR CONTRIBUTIONS

**Petra Kersten:** Data curation (equal); formal analysis (equal); investigation (equal); methodology (equal); validation (equal); writing – review and editing (equal). **Asja Vogt:** Data curation (equal); formal analysis (equal); investigation (equal); methodology (equal); validation (equal); writing – review and editing (equal). **Klaus Kohlmann:** Formal analysis (equal); investigation (equal); methodology (equal); validation (equal); writing – review and editing (equal). **Essetchi Paul Kouamelan:** Conceptualization (equal); supervision (equal); writing – review and editing (equal). **Thomas Mehner:** Conceptualization (equal); formal analysis (equal); funding acquisition (equal); methodology (equal); project administration (equal); resources (equal); software (equal); supervision (equal); validation (equal); writing – review and editing (equal). **Amien Isaac Amoutchi:** Conceptualization (equal); data curation (equal); formal analysis (equal); investigation (equal); methodology (equal); project administration (equal); software (equal); validation (equal); visualization (equal); writing – original draft (equal); writing – review and editing (equal).

## CONFLICT OF INTEREST

The authors have no conflicts of interest to declare.

## Supporting information


Appendix S1

## Data Availability

The sequences of microsatellite markers developed and used in this study have been submitted to Genbank (https://www.ncbi.nlm.nih.gov/genbank/).
