# Supplementary material for: Population genetics of the African snakehead fish Parachanna obscura along West Africa's water networks: Implications for sustainable management and conservation
Source: Ecol Evol. 2023 Jan 16;13(1):e9724. doi: 10.1002/ece3.9724 (PMC9842884; doi:10.1002/ece3.9724)
Supplement: Supplementary file 1 — Appendix S1 [file ECE3-13-e9724-s001.docx]

**APPENDICES**

**Appendix A**: Microsatellite markers developed and used in this study for the population genetic study of P. obscura with details on primer sequences, fluorescent dyes, repeat motifs and expected PCR amplification size.

| **Accession Number** | **Locus name** | **Forward sequence** | **Reverse sequence** | **Dye** | **Repeat motif** | **Expected size** |
| --- | --- | --- | --- | --- | --- | --- |
| OQ136685 | Para018 | CGTCCAGGAGTGGTCTTCAG | ACCTCATGATGGTGCTCTGG | BMN-6 | (AC)13 | 206 |
| OQ136686 | Para023 | AAACTGAACACAGGGCATGC | AGAGGTCTTGACATGATGTGGA | Cyanine 5 | (AC)12 | 296 |
| OQ136687 | Para025 | GGTGTTGTCACTTGTGACCC | AGCTGTGCAAATTACCTAGAAGC | Cyanine 5 | (AG)12 | 198 |
| OQ136688 | Para026 | CTGTTTGGTGTAGCACTAAAGGT | TTGATATCTGAACGGCTCCTTT | BMN-6 | (AC)12 | 190 |
| OQ136689 | Para027 | CCCGTGCAGTAAATGGATGAAG | GGATTCAAGGCTAGACTGAAAGA | Cyanine 5 | (AC)12 | 178 |
| OQ136690 | Para036 | AGCATGGTAATGAGCAGCTAA | TGTTTCAGAGCTAAGACGTGGT | BMN-6 | (AC)11 | 227 |
| OQ136691 | Para037 | CAGGTACAAATTGGTGGCCC | TACCCTGAAATGAGGCGCAG | DY-751 | (AC)11 | 222 |
| OQ136692 | Para038 | AAGCCACAGAACCAAAGCCA | GGACAAGTATCAGTGGCCGA | Cyanine 5 | (AC)11 | 205 |
| OQ136693 | Para039 | TCGTGTTAGGCGAGCATTCA | CTCGTAGCGTGAAGGGCTTT | DY-751 | (AG)11 | 198 |
| OQ136694 | Para040 | ACAAGCAAGTTTACATGCAGCA | ATGGAGGCAGATGATCCGCT | DY-751 | (AAG)11 | 192 |
| OQ136695 | Para041 | GGCACCGAAATGTCAGCAAC | ACAACAAACACTGAGCTCACTT | DY-751 | (AAT)11 | 128 |
| OQ136696 | Para042 | GGGAGGCGTACCAGCAAATA | CTGTTGCCACAGTTCCTTCA | BMN-6 | (AC)11 | 126 |
| OQ136697 | Para049 | GAGCATGTGGAGTAGGTGGG | ATTGAGGTGCTTTGGCCACT | BMN-6 | (AG)10 | 213 |
| OQ136698 | Para059 | GTCCCAGTACAACAGCGAGA | TCAGAGAAACCAGTGGACTGT | DY-751 | (AC)10 | 140 |
| OQ136699 | Para104 | TGTCAGCTTTCCAAGTACTGCA | TAATACCTCAGCTGCACCGG | Cyanine 5 | (ACC)8 | 211 |
| OQ136700 | Para107 | GTGGGCTCTGCACTAGTTGT | AGCCCAGACTAGAAGGAGCT | Cyanine 5 | (AC)8 | 206 |
| OQ136701 | Para110 | GGCGACACAAACATCTGAGC | TACCTGCGACCGGATGAGAT | DY-751 | (AC)8 | 198 |
| OQ136702 | Para128 | AATGACATAGACCGCTGGCC | AGCTGGGACAAGCCAAGATG | DY-751 | (AC)8 | 144 |
| OQ136703 | Para134 | ACAACAACTGCACACGCTTG | GGAGCGTGAATGCCTGTTCA | BMN-6 | (AC)8 | 139 |
| OQ136704 | Para136 | TGCTTAATCCTCTGCTGACGG | CTGTGTTGTTCCCACTTACGT | BMN-6 | (AGAT)8 | 137 |
| OQ136705 | Para137 | GCCCTCTGAGCGTGATCTTT | CCCATCTGTGACACTATGGTCT | DY-751 | (AAC)8 | 120 |

**Appendix B**: Composition of the PCR mix of the eight multiplex reactions performed in this study.

| **Multiplex** | **Product** | **Volume** | **Multiplex** | **Product** | **Volume** |
| --- | --- | --- | --- | --- | --- |
| **Multiplex 1** | Master mix | 5µl | **Multiplex 5** | Master mix | 5µl |
|  | dH2O | 4.32µl |  | dH2O | 4.63µl |
|  | Para136-F | 0.13µl |  | Para059-F | 0.065µl |
|  | Para136-R | 0.13µl |  | Para059-R | 0.065µl |
|  | Para025-F | 0.07µl |  | Para027-F | 0.04µl |
|  | Para025-R | 0.07µl |  | Para027-R | 0.04µl |
|  | Para137-F | 0.14µl |  | Para036-F | 0.08µl |
|  | Para137-R | 0.14µl |  | Para036-R | 0.08µl |
| **Multiplex 2** | Master mix | 5µl | **Multiplex 6** | Master mix | 5µl |
|  | dH2O | 4.55µl |  | dH2O | 4.62µl |
|  | Para042-F | 0.09µl |  | Para049-F | 0.07µl |
|  | Para042-R | 0.09µl |  | Para049-R | 0.07µl |
|  | Para038-F | 0.035µl |  | Para026-F | 0.12µl |
|  | Para038-R | 0.035µl |  | Para026-R | 0.12µl |
|  | Para039-F | 0.10µl |  |  |  |
|  | Para039-R | 0.10µl |  |  |  |
| **Multiplex 3** | Master mix | 5µl | **Multiplex 7** | Master mix | 5µl |
|  | dH2O | 4.59µl |  | dH2O | 4.67µl |
|  | Para128-F | 0.095µl |  | Para041-F | 0.12µl |
|  | Para128-R | 0.095µl |  | Para041-R | 0.12µl |
|  | Para107-F | 0.040µl |  | Para104-F | 0.045µl |
|  | Para107-R | 0.040µl |  | Para104-R | 0.045µl |
|  | Para134-F | 0.070µl |  |  |  |
|  | Para134-R | 0.070µl |  |  |  |
| **Multiplex 4** | Master mix | 5µl | **Multiplex 8** | Master mix | 5µl |
|  | dH2O | 4.49µl |  | dH2O | 4.68 |
|  | Para023-F | 0.05µl |  | Para037-F | 0.060µl |
|  | Para023-R | 0.05µl |  | Para037-R | 0.060µl |
|  | Para018-F | 0.11µl |  | Para040-F | 0.10µl |
|  | Para018-R | 0.11µl |  | Para040-R | 0.10µl |
|  | Para110-F | 0.095µl |  |  |  |
|  | Para110-R | 0.095µl |  |  |  |

**Appendix C:** Proportion of missing genotypes among the 21 microsatellites (columns) in the 15 locations Parachanna obscura (rows). No individual with more than two missing genotypes where found, thus, all the 259 Individuals used for subsequent statistical analyses.

|  | Para137 | Para136 | Para025 | Para042 | Para039 | Para038 | Para128 | Para107 | Para134 | Para023 | Para018 | Para110 | Para059 | Para027 | Para036 | Para049 | Para026 | Para041 | Para104 | Para037 | Para040 | Mean |
| --- | --- | --- | --- | --- | --- | --- | --- | --- | --- | --- | --- | --- | --- | --- | --- | --- | --- | --- | --- | --- | --- | --- |
| BAG | 0.000 | 0.000 | 0.000 | 0.000 | 0.000 | 0.000 | 0.000 | 0.000 | 0.000 | 0.000 | 0.000 | 0.000 | 0.000 | 0.000 | 0.000 | 0.000 | 0.000 | 0.000 | 0.000 | 0.000 | 0.000 | 0.000 |
| ABIN | 0.000 | 0.000 | 0.000 | 0.000 | 0.000 | 0.000 | 0.000 | 0.000 | 0.000 | 0.000 | 0.000 | 0.000 | 0.000 | 0.000 | 0.000 | **0.167** | **0.167** | 0.000 | 0.000 | 0.000 | 0.000 | 0.016 |
| KRINB | 0.000 | 0.000 | 0.000 | 0.000 | 0.000 | 0.000 | 0.000 | 0.000 | 0.000 | 0.000 | 0.000 | 0.000 | 0.000 | 0.000 | 0.000 | 0.000 | 0.000 | 0.000 | 0.000 | 0.000 | 0.000 | 0.000 |
| GLO | 0.000 | 0.000 | 0.000 | 0.000 | 0.000 | 0.000 | 0.000 | 0.000 | 0.000 | 0.000 | 0.000 | 0.000 | 0.000 | 0.000 | 0.000 | 0.000 | 0.000 | 0.000 | 0.000 | 0.000 | 0.000 | 0.000 |
| SASA | 0.000 | 0.000 | 0.000 | 0.000 | 0.000 | 0.000 | 0.000 | 0.000 | 0.000 | 0.000 | 0.000 | 0.000 | 0.000 | 0.000 | 0.000 | 0.000 | 0.000 | 0.000 | 0.000 | 0.000 | 0.000 | 0.000 |
| BAHO | 0.000 | 0.000 | 0.000 | 0.000 | 0.000 | 0.000 | 0.000 | 0.000 | 0.000 | 0.000 | 0.000 | 0.000 | 0.000 | 0.000 | 0.000 | 0.000 | 0.000 | 0.000 | 0.000 | 0.000 | 0.000 | 0.000 |
| SASB | 0.000 | 0.000 | 0.000 | 0.000 | 0.000 | 0.000 | 0.000 | 0.000 | 0.000 | 0.000 | 0.000 | 0.000 | 0.000 | 0.000 | 0.000 | 0.000 | 0.000 | 0.000 | 0.000 | 0.000 | 0.000 | 0.000 |
| NZOA | 0.000 | 0.000 | 0.000 | 0.000 | 0.000 | 0.000 | 0.000 | 0.000 | 0.000 | 0.000 | 0.000 | 0.000 | 0.000 | 0.000 | 0.000 | 0.000 | 0.000 | **0.083** | 0.000 | 0.000 | 0.000 | 0.004 |
| NZOB | 0.000 | 0.000 | 0.000 | 0.000 | 0.000 | 0.000 | 0.000 | 0.000 | 0.000 | **0.100** | 0.000 | 0.000 | 0.000 | 0.000 | 0.000 | 0.000 | 0.000 | **0.025** | 0.000 | 0.000 | 0.000 | 0.006 |
| KAN | 0.000 | 0.000 | 0.000 | 0.000 | 0.000 | 0.000 | 0.000 | 0.000 | 0.000 | 0.000 | 0.000 | 0.000 | 0.000 | 0.000 | 0.000 | 0.000 | 0.000 | 0.000 | 0.000 | 0.000 | 0.000 | 0.000 |
| SANP | 0.000 | 0.000 | 0.000 | 0.000 | 0.000 | 0.000 | 0.000 | 0.000 | 0.000 | 0.000 | 0.000 | 0.000 | 0.000 | 0.000 | 0.000 | 0.000 | 0.000 | 0.000 | 0.000 | 0.000 | 0.000 | 0.000 |
| SBR | **0.059** | **0.059** | 0.000 | 0.000 | 0.000 | 0.000 | 0.000 | 0.000 | 0.000 | 0.000 | 0.000 | 0.000 | 0.000 | 0.000 | 0.000 | 0.000 | 0.000 | **0.059** | 0.000 | 0.000 | 0.000 | 0.008 |
| KRINA | **0.026** | 0.000 | 0.000 | 0.000 | 0.000 | 0.000 | 0.000 | 0.000 | 0.000 | 0.000 | 0.000 | 0.000 | 0.000 | 0.000 | 0.000 | 0.000 | 0.000 | **0.026** | 0.000 | 0.000 | 0.000 | 0.003 |
| SIK | 0.000 | 0.000 | 0.000 | 0.000 | 0.000 | 0.000 | 0.000 | 0.000 | 0.000 | 0.000 | 0.000 | 0.000 | 0.000 | 0.000 | 0.000 | 0.000 | 0.000 | **0.200** | 0.000 | 0.000 | 0.000 | 0.010 |
| BIN | 0.000 | 0.000 | 0.000 | 0.000 | 0.000 | 0.000 | 0.000 | 0.000 | 0.000 | 0.000 | 0.000 | 0.000 | 0.000 | 0.063 | 0.000 | 0.000 | 0.000 | 0.000 | 0.000 | 0.000 | 0.000 | 0.003 |
| Total | 0.008 | 0.004 | 0.000 | 0.000 | 0.000 | 0.000 | 0.000 | 0.000 | 0.000 | 0.015 | 0.000 | 0.000 | 0.000 | 0.004 | 0.000 | 0.004 | 0.004 | 0.019 | 0.000 | 0.000 | 0.000 | 0.003 |

**Appendix D:** Allelic variability across the 21 microsatellites developed and used in this study. With N_A_: observed number of alleles, H_O_: observed heterozygosity_,_ H_S_: expected heterozygosity.

| Locus | H_O_ | H_S_ | N_A_ |
| --- | --- | --- | --- |
| Para018 | 0.15 | 0.53 | 6 |
| Para023 | 0.15 | 0.39 | 4 |
| Para025 | 0.04 | 0.50 | 3 |
| Para026 | 0.05 | 0.55 | 5 |
| Para027 | 0.42 | 0.67 | 12 |
| Para036 | 0.10 | 0.25 | 3 |
| Para037 | 0.17 | 0.51 | 5 |
| Para038 | 0.11 | 0.19 | 2 |
| Para039 | 0.06 | 0.21 | 3 |
| Para040 | 0.12 | 0.29 | 6 |
| Para041 | 0.13 | 0.41 | 3 |
| Para042 | 0.03 | 0.21 | 3 |
| Para049 | 0.19 | 0.44 | 3 |
| Para059 | 0.18 | 0.44 | 3 |
| Para104 | 0.31 | 0.50 | 4 |
| Para107 | 0.21 | 0.36 | 5 |
| Para110 | 0.25 | 0.66 | 7 |
| Para128 | 0.10 | 0.47 | 2 |
| Para134 | 0.07 | 0.28 | 4 |
| Para136 | 0.15 | 0.43 | 2 |
| Para137 | 0.12 | 0.39 | 2 |

**Appendix E:** Matrix of pairwise F_ST_ between the 15 *P.obscura* populations obtained using only the 19 microsatellite loci with low null allele frequencies.

|  | ABIN | BAG | BAHO | BIN | GLO | KAN | KRIN | KRINB | NZOA | NZOB | SANP | SASA | SASB | SBR |
| --- | --- | --- | --- | --- | --- | --- | --- | --- | --- | --- | --- | --- | --- | --- |
| BAG | 0.90 |  |  |  |  |  |  |  |  |  |  |  |  |  |
| BAHO | 0.83 | 0.95 |  |  |  |  |  |  |  |  |  |  |  |  |
| BIN | 0.70 | 0.83 | 0.74 |  |  |  |  |  |  |  |  |  |  |  |
| GLO | 0.82 | 0.96 | 0.66 | 0.75 |  |  |  |  |  |  |  |  |  |  |
| KAN | 0.80 | 0.92 | 0.85 | 0.63 | 0.85 |  |  |  |  |  |  |  |  |  |
| KRIN | 0.57 | 0.71 | 0.53 | 0.39 | 0.53 | 0.41 |  |  |  |  |  |  |  |  |
| KRINB | 0.58 | 0.79 | 0.56 | 0.43 | 0.55 | 0.50 | 0.01 |  |  |  |  |  |  |  |
| NZOA | 0.84 | 0.94 | 0.04 | 0.76 | 0.61 | 0.85 | 0.55 | 0.59 |  |  |  |  |  |  |
| NZOB | 0.89 | 0.93 | 0.07 | 0.84 | 0.59 | 0.88 | 0.64 | 0.72 | -0.03 |  |  |  |  |  |
| SANP | 0.60 | 0.76 | 0.52 | 0.54 | 0.57 | 0.54 | 0.32 | 0.33 | 0.54 | 0.65 |  |  |  |  |
| SASA | 0.84 | 0.93 | -0.02 | 0.78 | 0.55 | 0.85 | 0.56 | 0.62 | 0.01 | 0.05 | 0.55 |  |  |  |
| SASB | 0.86 | 0.94 | -0.07 | 0.79 | 0.61 | 0.87 | 0.58 | 0.64 | 0.07 | 0.11 | 0.58 | 0.00 |  |  |
| SBR | 0.85 | 0.93 | 0.22 | 0.77 | 0.61 | 0.85 | 0.56 | 0.62 | 0.08 | 0.09 | 0.54 | 0.11 | 0.25 |  |
| SIK | 0.58 | 0.89 | 0.69 | 0.61 | 0.66 | 0.68 | 0.47 | 0.46 | 0.71 | 0.80 | 0.46 | 0.72 | 0.77 | 0.70 |

**Appendix F**: P-values of deviations from Hardy-Weinberg equilibrium population after correction by the false discovery rate for multiple tests.

|  | BAG | ABIN | KRINB | GLO | SASA | BAHO | SASB | NZOA | NZOB | KAN | SANP | SBR | KRINA | SIK | BIN | N HWE per loci |
| --- | --- | --- | --- | --- | --- | --- | --- | --- | --- | --- | --- | --- | --- | --- | --- | --- |
| Para137 | 1 | 1 | 1 | 1 | 1 | 1 | 1 | 1 | 1 | 1 | 1 | 1 | 1 | 1 | 1 | 0 |
| Para136 | 1 | 1 | 1 | 1 | 1 | 1 | 1 | 1 | 1 | 1 | 1 | 1 | 1 | 1 | 1 | 0 |
| Para025 | 1 | 1 | 1 | 1 | 1 | 1 | 1 | 1 | 1 | 1 | 1 | 1 | 1 | 1 | 1 | 0 |
| Para042 | 1 | 1 | 1 | 1 | 1 | 1 | 1 | 1 | 1 | 1 | 1 | 1 | 1 | 1 | 1 | 0 |
| Para039 | 1 | 1 | 1 | 1 | 1 | 1 | 1 | 1 | 1 | 1 | 1 | 1 | 1 | 1 | 1 | 0 |
| Para038 | 1 | 1 | 1 | 1 | 1 | 1 | 1 | 1 | 1 | 1 | 1 | 1 | 1 | 1 | 1 | 0 |
| Para128 | 1 | 1 | 1 | 1 | 1 | 1 | 1 | 1 | 1 | 1 | 1 | 1 | 1 | 1 | 1 | 0 |
| Para107 | 1 | 1 | 1 | 1 | 1 | 1 | 1 | 1 | 1 | 1 | 1 | 1 | 1 | 1 | 1 | 0 |
| Para134 | 1 | 1 | 1 | 1 | 0.01 | 1 | 1 | 1 | 1 | 1 | 1 | 1 | 1 | 1 | 1 | 1 |
| Para023 | 1 | 1 | 1 | 1 | 1 | 1 | 1 | 1 | 1 | 1 | 1 | 1 | 1 | 1 | 1 | 0 |
| Para018 | 1 | 1 | 1 | 1 | 1 | 1 | 1 | 1 | 1 | 1 | 1 | 1 | 1 | 1 | 1 | 0 |
| Para110 | 1 | 1 | 1 | 1 | 1 | 1 | 1 | 1 | 1 | 1 | 1 | 1 | 1 | 1 | 1 | 0 |
| Para059 | 1 | 1 | 1 | 1 | 1 | 1 | 1 | 1 | 1 | 1 | 1 | 1 | 1 | 1 | 1 | 0 |
| Para027 | 1 | 1 | 1 | 1 | 1 | 1 | 0.02 | 1 | 1 | 1 | 1 | 1 | 1 | 1 | 1 | 1 |
| Para036 | 1 | 1 | 1 | 1 | 1 | 1 | 1 | 1 | 1 | 1 | 1 | 1 | 1 | 1 | 1 | 0 |
| Para049 | 1 | 1 | 1 | 1 | 1 | 1 | 1 | 1 | 1 | 1 | 1 | 1 | 1 | 1 | 1 | 0 |
| Para026 | 1 | 1 | 1 | 1 | 1 | 1 | 1 | 1 | 1 | 1 | 1 | 1 | 1 | 1 | 1 | 0 |
| Para041 | 1 | 1 | 1 | 1 | 1 | 1 | 1 | 1 | 1 | 1 | 1 | 1 | 1 | 1 | 1 | 0 |
| Para104 | 1 | 1 | 1 | 1 | 1 | 1 | 1 | 1 | 1 | 1 | 1 | 1 | <0.0001 | 1 | 1 | 1 |
| Para037 | 1 | 1 | 1 | 1 | 1 | 1 | 1 | 1 | 1 | 1 | 1 | 1 | 1 | 1 | 1 | 0 |
| Para040 | 1 | 1 | 1 | 1 | 1 | 1 | 1 | 1 | 1 | 1 | 1 | 1 | 1 | 1 | 1 | 0 |
| N HWE per population | 0 | 0 | 0 | 0 | 1 | 0 | 1 | 0 | 0 | 0 | 0 | 0 | 1 | 0 | 0 | 0 |

**Appendix G:** Output of BAYESCAN analysis for identifying candidate loci under selection. q-value: q-value for the model including selection. alpha: coefficient indicating the strength and direction of selection; A positive value of alpha suggests directional selection, whereas negative values suggest balancing or purifying selection. F_ST_: the F_ST_ coefficient averaged over populations. Significant q-value (lesser than 0.05) are bolded.

| **Locus** | **q-value** | **alpha** | **F_ST_** |
| --- | --- | --- | --- |
| Para137 | **0.048** | 0.88 | 0.78 |
| Para136 | 0.38 | 0.13 | 0.67 |
| Para025 | **0.00** | 1.72 | 0.87 |
| Para042 | 0.25 | 0.64 | 0.73 |
| Para039 | 0.45 | 0.06 | 0.65 |
| Para038 | 0.50 | 0.03 | 0.65 |
| Para128 | **0.01** | 0.99 | 0.79 |
| Para107 | 0.55 | 0.01 | 0.65 |
| Para134 | 0.57 | 0.00 | 0.65 |
| Para023 | 0.30 | 0.14 | 0.67 |
| Para018 | 0.13 | 0.37 | 0.71 |
| Para110 | 0.08 | 0.57 | 0.74 |
| Para059 | 0.21 | 0.41 | 0.71 |
| Para027 | **0.03** | -0.88 | 0.48 |
| Para036 | 0.53 | 0.01 | 0.65 |
| Para049 | 0.47 | 0.05 | 0.65 |
| Para026 | **0.00** | 1.38 | 0.84 |
| Para041 | 0.18 | 0.44 | 0.71 |
| Para104 | 0.41 | -0.10 | 0.63 |
| Para037 | 0.34 | 0.12 | 0.66 |
| Para040 | 0.59 | 0.00 | 0.64 |
